# 72957 Rethinking reconstructive strategies for complex cranial defects: A 10 year review of cranioplasty outcomes

**DOI:** 10.1017/cts.2021.588

**Published:** 2021-03-30

**Authors:** Edgar Soto, Carter J Boyd, Ryan Restrepo, Shivani Ananthasekar, Rene P. Myers

**Affiliations:** 1 University of Alabama School of Medicine; 2 New York University

## Abstract

ABSTRACT IMPACT: Up to 33% of patients of patients who undergo reconstruction have hostile defects with coexisting soft tissue and osseous defects due to prior radiation, prior failed cranioplasty or concurrent infections we seek to identify optimal strategies for these patients based on the experience of a southeastern tertiary referral center. OBJECTIVES/GOALS: Scalp and calvarial defects in patients may result from a number of etiologies including trauma, burns, tumor resections, infections, osteoradionecrosis, or congenital lesions. Our objective was to retrospectively evaluate the use of alloplastic reconstruction alongside autologous reconstruction for high risk cranial defects. METHODS/STUDY POPULATION: An IRB approved retrospective review of patients who underwent cranioplasty of a hostile site at a Southeastern tertiary referal center between January 2008 and December 2018 was performed. The patients were stratified into three groups based on the type of implant used: autogenous (bone), alloplastic (PEEK, Titanium, PMMA), or mixed (combination of both types of graft). The primary outcome metric was a complication in the year following cranioplasty, identified by flap or bone graft failure, necrosis, or infection. Statistical analysis included t-tests and chi-square tests where appropriate using SPSS. RESULTS/ANTICIPATED RESULTS: There were 43 total cases in this time period; 15 autogenous, 23 alloplastic, and 5 mixed. The purely autogenous group had the highest complication rate (85%) and the alloplastic group had the lowest complication rate (38%). When stratified by specific material used for reconstruction (15 bone, 14 PEEK, 10 titanium, and 5 PMMA), overall complication rate was statistically significant (p=0.009; chi square test) with PEEK implants having the lowest complication rate (21%). The analysis documented an overall complication rate that was statistically different between the three groups (p=0.012). DISCUSSION/SIGNIFICANCE OF FINDINGS: This analysis interestingly found that in the setting of hostile cranial defects, cranioplasties would benefit from the use of prosthetic implants instead of autologous bone grafts, not only for avoidance of donor site morbidity but also for decrease in overall complications.

